# General Medicine and Therapeutics

**Published:** 1895-01

**Authors:** 


### GENERAL MEDICINE AND THERAPEUTICS.

EDITED BY DR. LUTHER B. GRANDY.

### TREATMENT OF SOME FREQUENT MALADIES OF CHILDREN.

*Anemia.*—First, attention should be paid to hygiene and to regular feeding with nourishing and easily assimilable foods. Correct all intestinal disturbances. Before each feeding administer the syrup of the iodide of iron for fifteen days, followed for the next fifteen days by Fowler's solution, commencing with half drop for a child of two years.

From time to time suspend this treatment for a week and give—

Syrup of rhubarb,
Syrup of gentian, of each .... ......... ........ ....... .... ...f℥ iv.
Dessertspoonful for each dose.

Or of the following:

Tr. nux vomica.......................... ............................ *m* xv.

Tr. anise,

Tr. gentian,

Tr. cascarilla,

Tr. colombo, of each ..........................................fℨ i, *m* xv.

Dose.—From 5 to 10 drops.

If there is indigestion, administer after meals a small glass of this lemonade:

Acid hydrochloric................................................... *m* xv.
Syrup of lemon.......... .................. ........ ............... f℥ iii.
Distilled water.. ....................................... ..... .........O i.

If there are symptoms of rachitis, phosphated milk, phosphate of lime, or acid phosphate of lime in milk may be added to the above regimen, while stronger feeding also may be resorted to.

For anemia with scrofulosis, cod-liver oil in winter and syrup of the iodides in summer should be administered.

Syrup of the iodide of iron may be alternated with Fowler's solution at the same time that the oil is being given.

If there are symptoms of tuberculosis, to the foregoing treatment add this plan:

Upon a plate pour a mixture of—

Tar........................................ ℥ iiss.

Creosote........................................ ʒ iii. to ʒ vi.

and place over a night-lamp burning throughout the night in the sleeping-room of the infant.

*Anemia of Syphilitics.*—To the new-born give frequent small doses of mercury, and increase the dose; if the child is too feeble the medicine can be given by inunctions.

For anemia following malaria, administer wine of cinchona in doses from a coffeespoonful to a soupspoonful before meals for fifteen days; then for the next fifteen Fowler's solution, 1 to 3 drops.

In hemophilia or anemia from loss of blood, for the first few hours 1 to 2 drops of perchloride of iron every two hours, then three or our times a day.

For constipation at weaning-time, injections and glycerin suppositories, and before meals a spoonful of—

Syrup of rhubarb,
Syrup of gentian, of each, equal parts.

Or give every morning a coffeespoonful of—

Olei ricini,
Syrup of orange-flowers, of each........................................ f℥ ss.

Also, in the same dose—

Calcined magnesia,
Sulphur, sublimed,
Cream of tartar, of each........................................ ʒ i.
Essence of anise........................................ *m* iiss.

When the liver is sluggish, in the morning, once or twice a week, give the following in cachet, or in honey, or sugar water:

Calomel,
Scammony, of each........................................ gr. iii. to gr. v.

For a purgative:

Boiling water........................................ f℥ iii.

Manna in tears........................................ f℥ i.

Senna leaves........................................ ʒ i.

Powder of parched coffee........................................ ʒ iiss.

Strain and take during the day.

With atony of the intestines:

Tr. nux vomica.................................................... f℥ ss.
Tr. belladonna,
Tr. anise,
Tr. cascara, of each............................................f℥ iiss.
Given in water (8 to 12 drops) before meals.

For biliary calculus in infants, in the intervals, bicarbonate of sodium in grain doses for ten days. The following ten days—

Syrup of ether,
Syrup of turpentine, of each..................................f℥ iii.
A dessertspoonful before meals.

Twice a week add to the above a coffeespoonful of—

Sulphur sublimated,
Cream of tartar,
Magnesia, of each..............................................℥ v.
Essence of anise ...............................................*m* xv.
Before meals.

The following table of foods may prove useful:

*Foods Permitted.*—Milk, cream, and fresh cheese; soups; eggs; all meats in small quantities, especially chicken; legumes, well cooked and when green; potatoes; dried fruits, preferably cooked; marmalades and jams; cooked fish; bread in small quantity; alkaline waters.

*Foods not Permitted.*—Butter and fats, old cheese, pork, mushrooms and truffles, pastries and sweetmeats, dried legumes, sausages, asparagus and tomatoes, liquors, coffee, wines, and strong beers.

*For Nephritic Colic.*—Hot baths, afterwards blisters, hot poultices, or hot fomentations or stupes applied over the renal region; then administer every half-hour a dessertspoonful of the following mixture:

Antipyrin.........................................gr. viii. to gr. xv.
Chloroform water...............................f℥ i.
Lime-water........................................f℥ ii.
Syrup of ether,
Syrup of belladonnæ, of each..............f℥ iiss.
Syrup of orange-flowers ....................f℥ iiss.

If this is vomited, then give morphine hypodermically.—E. PÉRIER (*Revue Obstet. et Gynécol.*, August, 1894.)—*Therap. Gazette.*

### TREATMENT OF ALCOHOLISM BY STRYCHNINE NITRATE.

Breed concludes a paper in the *Medical News* on this subject as follows:

1. That we have in this drug a remedy that actually, for a period as yet undetermined, removes the desire for alcoholic stimulation in the chronic inebriate, and that without the least effort on his part.

2. A remedy that removes the distress and gnawing at the epigastrium, so common upon the withdrawal of alcohol.

3. A remedy that tones up the nervous system, allays the insomnia, the flighty and other bad feelings in the head, the mental disturbances, and the tremulous agitation and uncertainty of voluntary motions due to the withdrawal of stimulants.

4. A remedy that brings back the appetite and general physical vigor of the body.

5. A remedy that temporarily transforms a wholly demoralized creature into a man.

6. A remedy that is of great value in acute attacks of alcoholism.

7. Incidentally, a remedy that is an exceedingly good and safe heart-tonic.

8. More than all, a remedy that exerts a moral influence upon the patient, giving him what he had before wholly lost, to wit: hope, enthusiasm, self-confidence, and courage, where before was despondency, abandonment, and despair; a steady, straightforward gaze, and a bright, youthful, expression of the eye, which replaces the shamefaced, sneaking, apologetic air of total depravity of the chronic inebriate.

9. We have in the nitrate of strychnine not a remedy that will oblige a man to abstain from drink if he does not want to do so, and such subjects do not deserve one. From the results obtained by the gold cure, the silverash cure, the Keeley cure, etc., we may conclude that we have a remedy that is as efficient as any of these and much safer; a remedy, moreover, that is not secret, and can be used by men who know the action of drugs and can use them with discretion and safety to the patient.—*Gaillard's Med. Journal.*

### TREATMENT OF MIGRAINE.

Dr. H. Gradle contributes a useful paper to the *Medical News* of March 3, 1894, on this topic. After discussing the cure of the condition by the relief of ocular defects, he says of drugs, that he knows of none that will cure migraine except cannabis indica. According to his experience, from one-fourth to one-third of all patients are permanently benefited by its use. This applies equally to cases without a peripheral source of irritation as well as those with eye strain, who for some reason or other would not wear glasses. Seguin, who first popularized the use of Indian hemp in this disorder, advised its continued administration in every case, and claimed more or less complete relief in about one-half of his observations after several months' treatment.

When Gradle began the study of cannabis indica, about twelve years ago, many preparations in the market were inert. His first experience showed that in certain patients cannabis indica aborts each attack of migraine. If the first dose does not give complete relief, he gives another in six hours in slightly larger quantity. When moderate doses fail entirely, larger ones answer no better purpose. Very severe attacks are sometimes only relieved, but not entirely aborted.

Whenever hemp influences the individual attack, its continued use twice daily will usually protect the paient against the recurrence of the spells, and if persisted in for months will cure many, but not all of the cases. He does not believe it will prove of service in thoses cases in which it does not alleviate the pain at the time.

Other drugs recommended in the text-books for the cure of migraine, such as iron, strychnine, arsenic, and phosphide of zinc, he has used but little, and invariably with negative results.

If the individual attack does not yield to cannabis indica, it can be stopped almost invariably by antipyrin. Guarana has in former times more often failed than proved satisfactory in his practice. Bromides do not stop the attacks, although they may relieve the discomfort. Chloral aborts migraine only when it induces sleep in patients whose headache will yield to sleep. Morphine has no effect at all upon the attack. It only dulls the pain while the action lasts.—*Therapeutic Gazette.*

### THE ANTITOXIN TREATMENT OF DIPHTHERIA.

Katz has obtained excellent results with Aronson's antitoxin in diphtheria in the Emperor and Empress Frederick's Children Hospital, under Professor Baginsky's superintendence. In the last three years, 1891-1893, 1,081 cases of diphtheria have been treated in the hospital, of which 421, or 38.9 per cent. died, the mortality in the respective years being 32.5 per cent. in 1891, 35.4 per cent. in 1892, and 41.7 per cent. in 1893. From the commencement of this year up to March 14, 86 cases have been treated, with 38 deaths, or a mortality of 41.8. On the latter date the antitoxin treatment was commenced and employed in 128 out of 151 cases admitted to the hospital, 23 cases not being subjected to it for various reasons. In the 128 cases so treated only 17 deaths occurred, the mortality thus falling from 41.7 to 13.2 per cent. In all his clinical observations Dr. Katz is able to say that on no occasion could any deleterious effect be ever attributed to the employment of the antitoxin solution. If renal inflammation did occur, it followed quite a normal course, no bad effect could be observed upon the rhythm or tone of the heart and pulse.

Katz also inoculated 72 children exposed to the disease in order to determine the prophylactic value of the remedy. Of the 72 inoculated, only 8 were attacked, and so slightly as to be free from any evil consequences. The author states that in the records of the hospital there has never been so favorable a result in a series of diphtheria cases since the introducion of the antitoxin treatment.—*Univ. Med. Mag.*

---

### ALBUMINURIA, CASTS AND BRIGHT'S DISEASE.

Shattuck (*Boston Medical and Surgical Journal,* June 21, 1894,) has examined the urine of patients seeking his advice for various ailments. He has used boiling with additions of nitric acid and the Heller test, and considers them the most satisfactory.

He concludes his article as follows:

(1) Renal albuminuria, as proved by the presence of both albumin and casts, is much more common in adults quite apart from

Bright's disease or any obvious source of renal irritation than is generally supposed.

(2) The frequency increases speedily and progressively with increasing age.

(3) This increase with age suggests the explanation that the albuminuria is often an indication of senile change.

(4) Though it cannot be regarded as yet absolutely proved, it is highly probable that faint traces of albumin and hyaline and finely-granular casts of small diameter are often, especially after 50 years of age, of little or no practical importance.—*Univ. Med. Mag.*

---

### A GOOD COUGH MIXTURE.

The following appears to be highly recommended (Miss. *Med. Mo.*):

| | | |
|---|---|---|
| ℞ | Ammoniæ muriat | ʒ ijss. |
| | Spts. chloroform | ʒ vj. |
| | Tr. opii camph. | |
| | Tr. hyoscyami | aa ℥ j. |
| | Syr. tolu | q. s. ad ℥ iv. |

Sig. Two teaspoonfuls in water every two, three or four hours.

